# Binding in Haptics: Integration of “What” and “Where” Information in Working Memory for Active Touch

**DOI:** 10.1371/journal.pone.0055606

**Published:** 2013-02-06

**Authors:** Franco Delogu, Wouter M. Bergmann Tiest, Tanja C. W. Nijboer, Astrid M. L. Kappers, Albert Postma

**Affiliations:** 1 Utrecht University, Department of Experimental Psychology, Helmholtz Institute, Utrecht, The Netherlands; 2 Lawrence Technological University, Department of Humanities, Social Sciences and Communication, Southfield, Michigan, United States of America; 3 Utrecht University, Helmholtz Institute, Physics of Man, Utrecht, The Netherlands; 4 University Medical Center, Department of Neurology, Utrecht, The Netherlands; 5 Rudolf Magnus Institute of Neuroscience and Center of Excellence for Rehabilitation Medicine, University Medical Center Utrecht and Rehabilitation Center De Hoogstraat, Utrecht, The Netherlands; Radboud University Nijmegen, The Netherlands

## Abstract

Information about the identity and the location of perceptual objects can be automatically integrated in perception and working memory (WM). Contrasting results in visual and auditory WM studies indicate that the characteristics of feature-to-location binding can vary according to the sensory modality of the input. The present study provides first evidence of binding between “what” and “where” information in WM for haptic stimuli. In an old-new recognition task, blindfolded participants were presented in their peripersonal space with sequences of three haptic stimuli varying in texture and location. They were then required to judge if a single probe stimulus was previously included in the sequence. Recall was measured both in a condition in which both texture and location were relevant for the task (Experiment 1) and in two conditions where only one feature had to be recalled (Experiment 2). Results showed that when both features were task-relevant, even if the association of location and texture was neither necessary nor required to perform the task, participants exhibited a recall advantage in conditions in which the location and the texture of the target probe was kept unaltered between encoding and recall. By contrast, when only one feature was task-relevant, the concurrent feature did not influence the recall of the target feature. We conclude that attention to feature binding is not necessary for the emergence of feature integration in haptic WM. For binding to take place, however, it is necessary to encode and maintain in memory both the identity and the location of items.

## Introduction

Separate mechanisms have been shown to exist in humans for processing the *identity* and the *location* of objects. The functional and anatomical independence of the so-called “what” and “where” streams of processing has been repeatedly demonstrated in perception in different modalities [1], for vision; [2], [3], for audition; [4], [5], for touch; [6], for cross-modal touch-vision associations).

Converging evidence of feature-to-location binding, however, demonstrates that “what” and “where” information can be also associated through mechanisms of binding in perception [7] and working memory (WM) [8]. In a seminal work, Prabhakaran and colleagues investigated the neural substrate of WM binding within the prefrontal lobe [8]. In one of the experimental conditions, participants were asked to memorize four visually presented letters as well as the positions of the letters on the screen. Subsequently, participants were presented with a single target letter in a target location. The task required them to say “yes” (positive trials) whenever the target letter and the target location had been already presented in the previous display, regardless of whether the target letter was presented at that same location or at the location of another letter from the previous display. Results indicated faster and more accurate responses for positive trials in which the target letter was presented at the same location (i.e., intact condition), compared to positive trials in which the target letter was presented at the location of one of the other letters of the previous display (i.e., recombined condition). The authors reasoned that participants were faster in the intact condition because the target display matched the integrated representation in WM, and slower in the incongruent case where they had to reorganize the information in WM. They concluded that participants maintained information about the location and the identity of letters in an integrated fashion.

The association in memory between different dimensions of the stimulus is not necessarily bi-directional. Findings about non-mutual influences of one feature on the other show that, in some conditions, the encoding of a feature implicates the encoding of a second feature, but the encoding of the second feature does not implicate the encoding of the first [9], [10], [11], Experiments 3 and 4. In the visual domain, there is evidence that when humans intend to encode the identity of items, they also incidentally encode item position [9], [10] whereas when they have the intention to encode item position, they do not incidentally encode item identity [9]. A reverse effect is shown in an auditory working memory study by Maybery and colleagues [11]. They found that verbal identity encoding (spoken letters) influences the recall of the location of auditory sources, while the encoding of sound locations does not influence the recall of the identity of the items ([11], Experiments 3 and 4). Specifically, when participants were asked to memorize sound location only, the recall accuracy was impaired in trials in which the identity (spoken letter) was changed from encoding to retrieval. The authors interpreted this result as evidence of a primacy of identity over location in the representation of sounds in working memory. The contrasting results between vision and audition support the idea that mechanisms of feature-location binding can vary as a function of input modality [11]. As such, it is highly interesting also to study binding mechanisms in other than visual and auditory sensory modalities. Concerning this, a promising direction of research is the haptic domain, but, remarkably, the analysis of the interaction between item identity and item location in active touch has been almost entirely neglected by research so far.

The few studies that approached feature-to-location binding in haptics did not directly deal with working memory, but aimed instead at testing “what” versus “where” interference effects in perceptual tasks. For example, Purdy, Lederman and Klatzky [12] asked participants to perform a feature detection task (i.e., roughness, edges, relative orientation or left/right oblique orientation) of tactile stimuli presented on different fingers of each hand. They found that participants showed longer reaction times (RTs) when they were requested, not only to recall information about these features, but also to report on which finger the stimulation was presented, compared to a condition in which no spatial recall was required. They concluded that location of tactile items in body space is not automatically processed within object features [12]. Purdy et al.’s finding only pertains to the influence of spatial encoding on the detection of the identity of the tactile stimulus. It does not say anything about the influence of the identity of the stimulus on a spatial task. More recently, Chan and Newell ([6], Experiment 1) addressed the topic of the bi-directional dissociability between “what” and “where” processing in the haptic domain. They used a dual-task interference paradigm in which participants were required to perform a primary recognition task while also performing an interference task. They tested conditions in which both primary and interference tasks were either in the “what” domain (e.g. shape and roughness) or in the “where” domain (e.g. location and orientation) and conditions in which the primary and the interference task were in different domains (e.g. shape and orientation or location and roughness). Their results indicated that when the interference and the primary task pertained to the same domain, participants found more difficult to perform the primary task, both in the “what” (shape) domain and in the “where” (location) domain. Chan and Newell concluded that, as in the visual system, also in the tactile systems, information is processed independently for recognition and for spatial localization [6].

The two abovementioned haptic studies show evidence of independent processing of spatial and identity information. The identity-location dissociability in the tactile domain contrasts with evidence of the automaticity of feature-to-location binding in visual [8] and in auditory WM [11], Experiments 1 and 2. However, the evidence of dissociation of location and identity in haptics is found in perceptual studies only by means of experimental designs that are markedly different from the ones used in WM binding studies. A direct test of identity-location binding in haptic working memory can provide important indications about the mechanism of haptic WM and about the presence of common cross-modal mechanisms in feature binding in WM in general.

In this study, we conducted two experiments aimed at directly testing whether the texture and the location of haptically explored objects are maintained in an integrated or in an independent fashion in WM. In Experiment 1, we associated location and texture in a conjunct condition where both features were relevant for the task. The main focus of Experiment 1 was to test whether an advantage in recognition would be observed for the intact probes over the recombined ones, consistent with a hypothesis of association in WM of the identity and the location of the tactile stimuli. In Experiment 2, we tested location and texture in two separate tasks. The second experiment required recognition judgments that were focused on either the identity or the location of the tactile items. These conditions allowed us to test whether the influences of the unattended feature - either the identity or the location - on the target feature are symmetric or asymmetric and whether identity-location binding takes place even when it is not necessary to memorize both features.

## Experiment 1

We used a modification of the experimental paradigm proposed by Prabhakaran and collaborators [8]. Since shape exploration is known to be particularly sensitive to orientation and variation of the external and/or body-centered frames of reference [13], we preferred to use textures instead of shapes to operationalize the feature identity.

Participants were presented in a learning phase with different stimuli varying in texture (T) and location (L): for example T_1_L_1_, T_2_L_2_, T_3_L_3_. The learning phase was followed by a test phase in which a single probe stimulus was presented for immediate recall. The task required indicating whether both the texture and the location of the probe stimulus were presented in the learning phase. The following probe conditions were tested: an old texture in its original location (e.g. T_2_L_2_), an old texture in the location of another old texture (e.g. T_2_L_3_), a new texture in the location of an old texture (e.g. T_new_L_2_), an old texture in a new location (e.g. T_2_L_new_) and a new texture in a new location (e.g. T_new_L_new_). The critical comparison was between *intact probes* (e.g. T_2_L_2_), where the association of the features is preserved between one of the stimuli of the learning sequence and the probe stimulus, and *recombined probes* (e.g. T_2_L_1_), where both a texture and a location used in the learning sequence were re-presented in the probe stimulus, though in a new combination. Since the task did not require associating texture and location, we reasoned that, if the two features were encoded independently, participants should show equivalent proficiency in responding to intact and recombined probes. Otherwise, if texture and location were integrated into multi-featured representations in WM, then intact probes should be recognized with greater ease than recombined probes. This is because an intact probe would match precisely the multi-featured representation of one of the learned stimuli, whereas a recombined probe would provide only a partial match to the representations of two learned stimuli.

### Methods

#### Ethics statement

Our research involved healthy human participants in non-clinical behavioral testing. The experiments were conducted in agreement with the ethics and safety guidelines of Utrecht University, which are based on the Declaration of Helsinki. A written informed consent was obtained from all participants. Under the advice of the WMO Advisory Committee of the Faculty of Social and Behavioral Sciences at Utrecht University, we decided not to submit our study for approval to the Medical Review Committee (METC) of the Utrecht Medical Center (UMC), as an explicit approval was not necessary for studies of this kind.

#### Participants

Twenty right-handed students of Utrecht University (mean age: 24.8 (SD = 4.0), 14 females) participated in the experiment in exchange for course credits or a small amount of money. All participants self-reported normal hearing, normal touch and normal or corrected-to-normal visual acuity.

#### Materials and apparatus

Two sets of 12 flat wooden squares of 10×10 cm were used. To the top of each square a specific material (fabric, stone, glass, plastic, wood) was attached which, when touched, was distinguishable from the others by its texture. Textures were selected from a set of 124 stimuli used in a previous study [14]. In that study, participants were asked to group together textures that felt similar. By counting the number of times that each combination of two textures occurred in the same group, similarity values for all combinations of two textures were obtained. From the original set, 24 stimuli were dropped because they had easily identifiable characteristics, making them unsuitable for the present study. For the remaining 100 textures, the average total similarity (i.e., sum of the similarity values of all possible pairs within the subset) of a large number of randomly selected subsets of 12 textures was calculated. Then, two non-overlapping subsets of 12 textures were selected that were as close as possible to this average total similarity. The total similarity of the two subsets was equal. This level of similarity was chosen in order to avoid floor or ceiling effects. By comparing the similarity judgments from several sub-groups of participants, it was found that different people assess the similarity within the chosen subsets of stimuli in a very comparable fashion.

**Figure 1:**
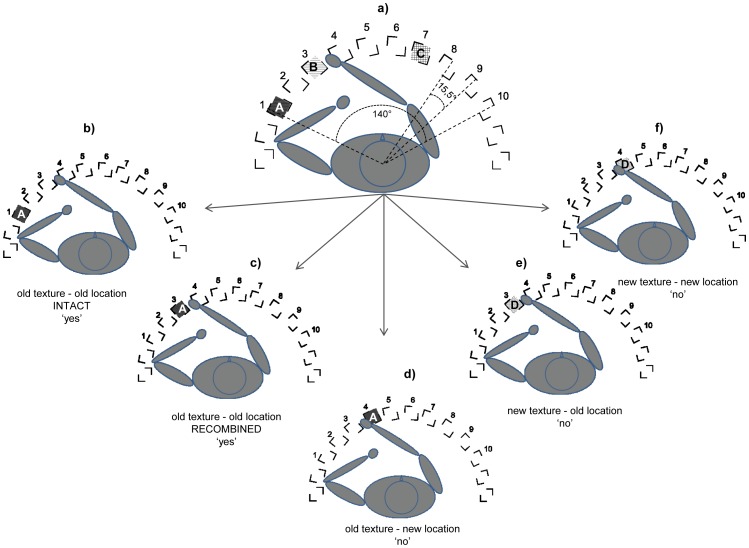
Setup and procedure of Experiment 1. During the learning phase (a) participants actively explore three textures (for example A, B, C) placed at locations (for example 1, 3, 7), and are asked to memorize both locations and textures. Then, during the probe phase, in five different probe conditions (b, c, d, e, f), they have to say “yes” if both the location and the texture of the probe item have been presented in the study phase.

An arc-shaped exploration space subtending an angle of 160 degrees was arranged on a table in front of the blindfolded participant (see top part of Figure 1). On this space, L-shaped aluminum bars defined 14 distinct positions, approximately equidistant from the participant’s body midline, in which textured blocks could be individually placed. Only the 10 central positions, defining a total angle variation of 140 degrees, were used as experimental positions, whereas the 2 rightmost and the 2 leftmost remained empty to avoid making the extreme positions (i.e., 1 and 10) easier to localize. A resting position where participants placed the middle finger of their right hand when they were not exploring was located in the space between the exploration space and the participant’s body, at zero degrees of azimuthal separation from the midsagittal plane. The resting position was marked by a rubber dot on the table surface.

#### Task and procedure

Before entering the experimental room, all participants were instructed about the spatial arrangement of the setup and trained in the exploration task with practical examples of texture exploration. Next, participants were blindfolded and entered the experimental room, which they had never seen before. During the experiment, they sat in a non-rotating chair facing the center of the exploration space. They started each trial with the middle finger of their right hand on the resting position in front of them. In order to prevent the influence of auditory localization cues produced by the experimenter when placing squares in position and by participants when rubbing on textured surfaces, participants wore noise-canceling headphones (Bose QuietComfort 15). Each trial included a learning phase and a probe phase. In the learning phase, the experimenter took three items from a set of twelve different textures and placed them in three of ten possible positions in the exploration space. The selection of textures, of positions and of their combination was quasi-random; in order to avoid confusion of items and positions belonging to different trials, a constraint was applied to the randomization process of stimuli and position that assured that textures and positions used in one trial could not be used in the following one. The list of positions and textures to be used in each trial of a block was displayed in a computer screen. The list of position-texture pairings was also used by the experimenter to record the participant’s responses in a digital file.

In the learning phase, when verbally prompted, participants moved from one of the extreme positions – either the leftmost or the rightmost in the exploration space – towards the other extreme position until they found the first of the three stimuli in the learning sequence. They could feel its texture by rubbing one or more fingers over the surface of the square. Although exploration time was not fixed, participants were instructed to explore the texture rapidly. After exploration of the first texture they proceeded along the exploration space in the same direction they started, seeking a new texture to explore. Participants were not allowed to go back and re-explore previously touched textures. The experiment was divided into two sessions. In the first session, half of the participants started their exploration from the rightmost position proceeding leftward and the other half started from the leftmost position proceeding rightward. The direction of exploration was reversed in the second session. During exploration, participants performed an articulatory suppression procedure by sub-vocalizing the word ‘cola’ in order to prevent verbal recoding and rehearsal. When all the three textures had been explored, participants returned to the waiting position. Subsequently, in the probe phase, a single probe texture was placed in one of the ten positions of the exploration space. The experimenter was trained to remove the three textures used in the learning phase and to place the probe texture on the target position from the exploration space as rapidly as possible, but without interfering with any movement of the participant during exploration. As soon as the participant reached the resting position after the learning phase exploration, the probe texture had already been placed in the correct position and the participant could be immediately prompted to start the probe phase. This way, the delay between learning and probe phases was always equivalent for all trials and for all participants. In the probe phase, the single texture present in the exploration space might or might not have been already explored in the previous display. The position might or might not have been used in the learning phase. Participants haptically rescanned the exploration space with the same direction of exploration used for the learning sequence until they found the probe square. For each trial, one of the probe types shown in Figure 1 was presented. Participants had to provide a ‘yes’ or a ‘no’ response according to the following conditions. Two probe types require a ‘yes’ response: intact probes, where a texture from the learned sequence is presented in its original location, and recombined probes, where a texture from the learned sequence is presented in a location used for a different texture in the learned sequence. Three probe types require a ‘no’ response: *new texture – new location* where neither the texture nor the location of the probe were not included in the learned sequence, *old texture – new location* and *new texture* – *old location* in which either the texture or the location were new while the other feature remained unchanged between learning and probe phase. The amount of time to produce a response was unconstrained. Although participants were not forced to answer within a specific time limit, they tended to respond with comparable delays, which were typically in the range of 3 to 5 seconds from the first contact with the probe texture.

After responding, participants replaced their middle finger in the resting position waiting for the next trial. Eighteen trials per probe type (90 in total) were presented in two sessions of 45 trials. In order to attenuate the effects of learning, different sets of stimuli were used in the first and in second sessions. The experiment lasted about 1 hour and 30 minutes.

### Analysis

The proportion of correct ‘yes’ and ‘no’ responses were calculated and signal detection theory (SDT) was employed to calculate sensitivity (i.e., d-prime) and the presence of a bias in the response (i.e. criterion). *d'* was calculated according to the following formula: *d'* = Z*_H_* – Z*_FA_* where *H* is HITS (proportion of ‘yes’ responses when both the texture and the location of the probe were present in the learning sequence), *FA* is FALSE ALARMS (proportion of ‘yes’ responses when either the probe texture or the probe location or both were not presented in the learning sequence) and the function Z*_p_*, with *p* ∈ [0,1], is the inverse of the cumulative Gaussian distribution. The criterion was calculated as follows: *C* = −0.5[Z*_H_*+Z*_FA_*]. In addition to the signal detection analysis, two separate Analyses of Variance (ANOVA) with respectively positive and negative probe types as single factors were performed on the percentages of correct responses. The choice to separate the analyses of positive and negative probes was led by different reasons. First: we reckoned that positive and negative probes inform about different aspects of our theoretical questions. In fact, data from positive probes tell us whether or not recall is easier for intact than for recombined probes. Data from negative probes, instead, inform us about the relative weight of identity and location in the correct rejection of a negative probe. Second: by splitting the analysis we did not lose information about sensitivity as we already obtain a measure of sensitivity from the SDT analysis. Third: a previous study which we wanted to compare to ours [11] used the same analysis for an analogous experimental procedure in the auditory domain. For post-hoc analyses, Bonferroni Correction was applied to pairwise comparisons.

### Results

Concerning SDT, the overall *d'* was 1.34, suggesting that participants were rather sensitive to variations in the probe stimuli. Moreover, the value of the criterion *C* was 0.03, which was not significantly different from zero: *t*(19) = 0.55, *p* = 0.59. This indicated that there was neither a bias toward a liberal approach (i.e., more ‘yes’ responses) nor a bias toward a conservative approach (i.e., more ‘no’ responses).

**Figure 2:**
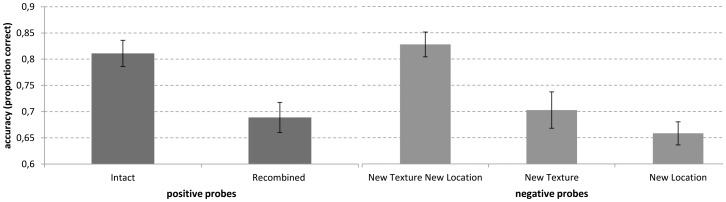
Proportion of correct responses as a function of the probe condition. Error bars indicate standard errors.

Accuracy in all probe conditions is summarized in Figure 2. The ANOVA with positive probe types revealed that the accuracy in the intact probe condition (81%) was significantly higher than the accuracy in the recombined probe condition (69%): *F*(1, 19) = 13, *p* = 0.0019, η**_p_^2^** = 0.41. The ANOVA with negative probe types (i.e., new, new-location, new-texture) indicated a significant effect of probe type *F*(2, 18) = 27, *p*<0.001, η**_p_^2^** = 0.75. Pairwise comparisons specified that the new texture – new location probe condition (83% correct) was significantly more accurate than the old texture - new-location probe condition (66% correct) (*p*<0.001). The new texture – new location probe condition was also significantly more accurate than the new texture – old location probe condition (70% correct) (*p*<0.001). Finally, no significant difference between the old texture – new location probe and the new texture – old location probe condition was found (*p* = 0.64).

In order to test the presence of serial position effects, we compared the frequency of correct recalls of intact probes which were previously explored in the learning phase as first (81% of correct responses), as second (84% of correct responses) or as third item (79% of correct responses) in the learning phase sequence. The Chi Square analysis indicated that the percentage of correct responses did not differ as a function of the serial position in which the probe was presented in the learning phase, χ^2^(2, N = 360) = 1.63, *p* = .44.

### Discussion

In Experiment 1, participants were presented with three stimuli varying in texture and location followed by a single probe stimulus. They were required to indicate whether the texture as well as the location of the probe stimulus had been presented in the learning sequence. Concerning general distinguishability, SDT analysis showed that participants were highly accurate in recalling textures and locations and did not show any response bias. Concerning feature binding, we compared accuracy in intact and recombined probes. In intact probes, both the texture and the location of one of the stimuli of the learning sequence were also presented in the probe stimulus. In recombined probes, the texture of one of the stimuli of the learning sequence and the location of another stimulus of the same list were recombined in the probe stimulus. Results indicated that, even if the combination texture and location was not relevant for the task, intact probes were recognized more accurately than recombined probes. This finding supports the hypothesis of an automatic integration of texture information and spatial location in WM representation and it is consistent with earlier evidence of feature-to-location binding in visual [8] and in auditory WM ([11], Experiments 1 and 2).

We also compared the accuracy in three different types of negative probes. Probes in which neither texture nor location were previously presented in the learning sequence were correctly rejected more often than probes in which only texture or only location were new. Importantly, the result of this comparison between negative probe conditions indicates that the relative weight of spatial and texture information is comparable and, therefore, that both location and texture equally contribute to memory recall. In sum, Experiment 1 revealed that when memory of both features is needed for an accurate response, item location and texture are represented in an integrated fashion in haptic WM.

Finally, we tested whether the serial order of the item during encoding influenced accuracy of recall. We did not find any difference in the recall performance for probes that were previously encoded either as the first, second or third item during the learning phase. We can therefore exclude the presence of serial order effects in the memory representation of brief (3 items) sequences of tactile items.

## Experiment 2

Since in Experiment 1 memory of both features was required to formulate a correct response, it was not possible to isolate the separate contributions of texture and location to the combined response. More importantly, it was also not possible to determine if texture-location binding takes place automatically even when it is not necessary to memorize both features. In order to clarify this issue, in Experiment 2 we investigated binding effects when only one of the two features was relevant for the memory task. Specifically, we used the same five probe conditions of Experiment 1 in two tasks in which memory for location and memory for texture were tested separately.

### Methods

#### Participants

Twenty right-handed students of Utrecht University (mean age: 22.6 (SD = 2.5), 14 females) participated in the experiment in exchange for course credits or a small amount of money. All participants self-reported normal hearing, touch and normal or corrected to normal visual acuity. Informed consent was obtained from all participants. None of the participants of Experiment 2 had taken part in Experiment 1.

#### Stimuli and apparatus

Stimuli and apparatus were the same as the ones used in Experiment 1.

#### Task and procedure

The main difference with Experiment 1 was the presence of two distinct and independent tasks for location and for texture discrimination. Four sessions of 30 trials each were performed with 12 trials per probe type. In two blocks, participants were required to memorize and recall only the position of the stimuli, whereas in the other two blocks, they were told to attend to the textures of the stimuli only. Two directions of exploration (from left to right and from right to left) were required in both tasks. The order of presentation of the two tasks (texture and location) and the direction of exploration (from right to left and from left to right) were counterbalanced between participants. Analogously to Experiment 1, an articulatory suppression task was required during exploration. Supplementary tasks were added during the learning phase to guarantee the perceptual processing of the task-irrelevant dimension. Specifically, in the location blocks, participants were required to say aloud “rough” or “smooth” according to their perception of each item along the dimension roughness/smoothness. Analogously, when performing the texture blocks, participants judged the location of each item, saying aloud “right” or “left” according to the position of the item with respect to their midsagittal plane. This way, we were sure that the information about the task-irrelevant feature was perceptually processed, although not necessarily maintained in WM. Two probe types, *intact* and *recombined,* in which both the texture and the location of the probe were included in the learning sequence, required always a ‘yes’ response, irrespective of the task. One probe type, *new texture – new location,* where both the texture and the location of the probe were not included in the learning sequence, required always a ‘no’ response irrespective of the task. Two probe types required a ‘yes’ response in one task and a ‘no’ response in the other task: *new location – old texture* (‘yes’ in the spatial task, ‘no’ in the identity one) and *old location – new texture* (‘yes’ in the identity task, ‘no’ in the spatial one). Each participant completed 4 sessions of 30 trials for a total duration of 1 hour and 45 minutes, including breaks between sessions.

### Analyses

Concerning SDT analysis, analogously to Experiment 1, we calculated *d'* and the criterion for both the texture and the location tasks. We ran two repeated-measures ANOVAs to measure the influence of the task on the d-prime and on the criterion, respectively. Regarding the role of the probe conditions, we conducted separate ANOVA analyses for the location task and for the texture task, as well as for positive and negative probes.

### Results

The ANOVA between d-primes of the texture (*d'* = 1.49) and location (*d'* = 1.54) tasks, indicated that participants were comparably sensitive to variations in the texture and in the location of the stimuli: *F*(1, 19) = 0.079, *p* = 0.22, *η_p_* ^2^ = 0.009. Concerning the response bias, one-sample *t*-tests indicated that the criterion in the texture task (*C* = 0.17) and in the location task (*C* = 0.13) were both significantly different from zero: *t* (19) = 2.6, *p* = 0.016 for the texture task and *t* (19) = 2.4, *p* = 0.029 for the location task. These criterion values indicate a bias towards a positive response, namely the tendency to produce ‘yes’ responses, which is often termed a “liberal approach”. The ANOVA between criteria of the location and of the texture task were not significantly different: *F*(1, 19) = 0.25, *p* = 0.37, η**_p_^2^** = 0.013.

**Figure 3:**
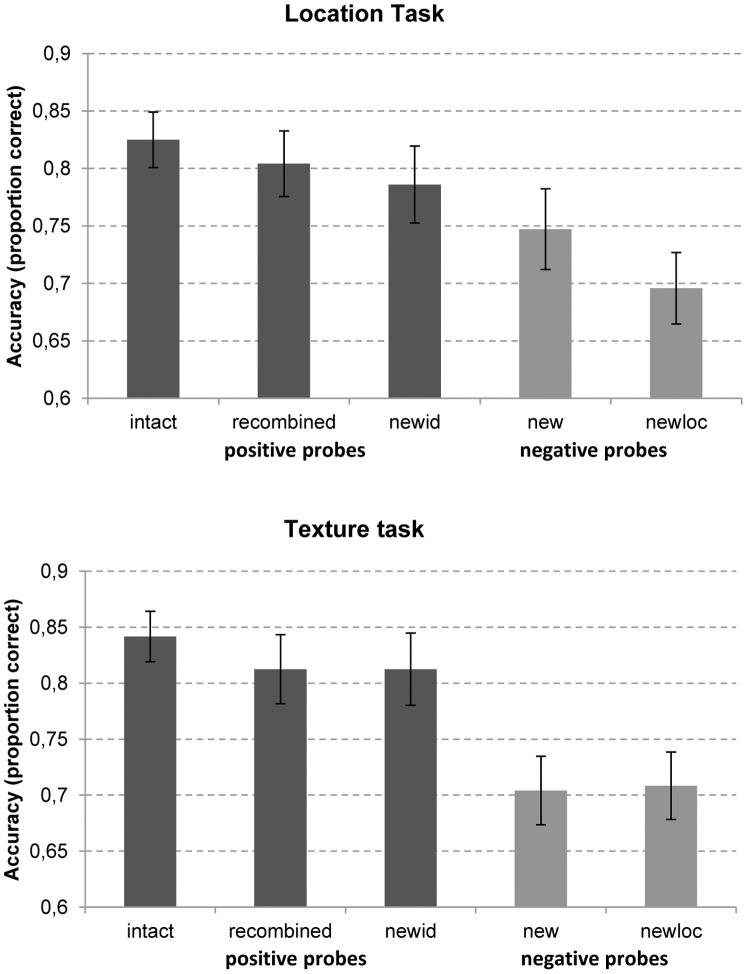
Proportion of correct responses as a function of the task and probe condition. Error bars indicate standard errors.

Results about accuracy in the different conditions of the location and the texture tasks are shown in Figure 3. In the location task, the ANOVA between positive probes, i.e., intact (82% correct responses), recombined (80% correct) and new texture – old location (78% correct) showed no difference between the three different conditions: *F*(2, 18) = 0.58, *p* = 0.57, *η* ***_p_*** **^2^** = 0.060. Also, no difference was found in the comparison of the two negative probe conditions, i.e., new texture - new location (75% correct) and old texture – new location (79% correct): *F*(1, 19) = 1.1, *p* = 0.31, *η* ***_p_*** **^2^** = 0.055. Analogously, in the texture task, the comparison between the three different positive probe conditions, i.e., intact (84% correct responses), recombined (81% correct) and old texture – new location (81% correct responses) showed no difference between conditions, *F*(2, 18) = 0.67, *p* = 0.52, *η* ***_p_*** **^2^** = 0.069. Also, no difference was found between the two texture negative probes, i.e., new texture – new location (70% correct) and new texture – old location (71% correct): *F*(1, 19) = 0.012, *p* = 0.91 *η* ***_p_*** **^2^** = 0.001.

We performed additional analyses in order to measure the influence of intervening factors, like the position of the probe item along the exploration arch and the direction of exploration. Specifically, we wanted to verify whether certain positions along the exploration arch would be easier to recall and whether lateralization could influence item representation in WM. An ANOVA with probe location eccentricity (5 levels: positions 1 and 10, 2 and 9, 3 and 8, 4 and 7, 5 and 6) and task (2 levels: location, texture) as factors showed no effect of probe location, *F*(3.2,16) = 1.1, *p* = 0.39, η**_p_^2^** = 0.22, no effect of task *F*(1,19) = 3.29, *p* = 0.08, η**_p_^2^** = 0.15, but a significant interaction between eccentricity and task: *F* (3.6,16) = 3.3, *p* = 0.023, η**_p_^2^** = 0.172. The post-hoc analysis (i.e., Bonferroni) indicated that participants in the location task only were more accurate (*p* = 0.04) in the extreme positions (1 and 10) than in the positions next to the extreme positions (2 and 9). A comparison was also made between recall accuracy of probe items located in position 1 and probe items in position 10, which are the most dissimilar in terms of movement and positioning of hand, arm and torso during exploration. Paired-samples t-tests indicated no difference between the two conditions, neither in the location task t(19) = 0.8, *p* = 0.43 nor in the texture task t(19) = −0.31, *p* = 0.76.

Concerning the influence of the direction of exploration, a two-factors ANOVA with the direction of exploration (2 levels: left, right) and task (2 levels: location, texture) showed no effect of direction: *F*(1,19) = 2.4, *p* = 0.1, η**_p_^2^** = 0.11, no effect of task (*F*(1,19) = 0.14, *p* = 0.28, η**_p_^2^** = 0.007) and no interaction between direction and task *F* (1,19) = 0.076, *p* = 0.21, η**_p_^2^** = 0.004.

### Discussion

In Experiment 2, where the spatial and the identity tasks were separated, accuracy in the recall of both the location and the texture of items did not vary as a function of the probe type. This result indicates that when one of the two features is task-irrelevant, the representation of the two features is not necessarily integrated in WM. This lack of integration is not caused by a lack of processing, since participants were forced to process the task-irrelevant feature in supplementary tasks during encoding. We interpret this result as evidence that information can be discharged from WM maintenance when it is not relevant for the task. Notably, previous studies in other sensory modalities have found that variations in the task-irrelevant dimension can affect the recall of the target dimension. For example, Maybery and colleagues ([11], Experiments 3 and 5) showed a non-symmetrical interaction between the identity and the location of auditory stimuli in which identity affected location recall but location did not affect identity recall. The authors concluded that identity is a paramount feature in auditory processing, whereas location is a subordinate one. Evidence of a cross-domain influence of a task irrelevant feature was also found in the visual domain. Interestingly, cross-domain effects in vision seem to proceed in the opposite direction, with item location influencing item identity [10], [9]. In particular, Olson and Marshuetz demonstrated that memory for item identity is influenced by the incidental encoding of item position [10]. In this case, the inverse influence, i.e., of identity on location, was not tested. In vision, the influence of location on identity and the lack of influence of identity on location were shown by Köhler and colleagues [9]. The difference between our results and the abovementioned studies in hearing and vision are probably related to the relative weights of identity and location in encoding. In our study, the two features were balanced in terms of encoding accuracy. We suggest that the influence of identity on location ([11], Experiments 3 and 4) and of location on identity [10], [9] can be explained as mere consequences of task difficulty instead of as the result of asymmetrical binding and sensory dominance on specific feature processing. More generally, it is safe to claim that, since previous studies employed dissimilar paradigms, procedures and stimuli, existing assumptions about the presence/absence of asymmetrical binding between features and about its relationship with sensory modalities are still not conclusively proven or disproven. However, on the basis of our data, we can maintain that in haptic WM, neither texture nor location is a prevailing feature.

Finally, apart from a small advantage in the recall of probe items located either in the rightmost or in the leftmost positions, we did not find any influence of the biomechanical features of the haptic exploration on the memory measures. These data are somewhat surprising. In fact, item exploration was associated with movements and positions of the hand, the arm and the torso that differ sensibly according to the position of the item in the exploration arch. Consequently, it was to be expected that different body positions could be used as cues for item recall. Remarkably, we did not find any influence of probe item position on the memory tasks. We speculate that the absence of influence of the dynamics of the motor exploration could be due to an allocentric recoding of the location of items [15]. It is likely that items are represented in mental maps of textures in space in which the specificity of the motor experience during exploration is not part of the memory trace. More studies are needed to test this assumption.

### Cross-experiment Comparison

In order to verify whether the simultaneous encoding of the two features impairs sensitivity to variation in a single feature, we ran two between-subjects ANOVAs comparing d-prime in the conjunction task (Experiment 1) to d-prime in the location-only task and in the texture task, respectively (Experiment 2). Notably, results indicated no difference between *d'* in the conjunction and in the location-only tasks (*F*(1, 19) = 2.2, *p* = 0.15), **η_p_^2^** = 0.10) and between the conjunction and in the texture condition (*F*(1, 19) = 0.86, *p = *0.37), **η_p_^2^** = 0.043). If binding would cause an impairment of sensitivity, we would expect a worse performance in the conjunction condition than in the single-feature conditions. This was not the case, since we did not find any negative effect of binding on sensitivity. We interpret this result as further proof of the automaticity of binding between texture and location in haptics.

## General Discussion

In Experiment 1, we showed that the accuracy in the conjunct recall of texture and location of a sequence of haptically explored items varies as a function of the probe type. More specifically, even if the combination of texture and location was not relevant for the task, intact probes were recognized more accurately than recombined probes. We interpreted this data as evidence of binding between spatial and texture information in WM, as already observed in vision [8] and audition [11] with similar paradigms. In Experiment 2, where either texture only or location only was relevant for the task, we found that accuracy in the recall of location and texture does not vary as a function of the probe type. We interpreted this result as evidence that both the location and the identity must be maintained in memory for identity-to-location binding to take place. Previous studies in vision and hearing have shown influences of the task-irrelevant feature on the recall of the target feature. More specifically, non-symmetrical cross-feature influences of identity on location in hearing ([11], Experiments 3 and 4) and of location on identity in vision have been found [9]. By contrast, in haptics, we did not find any evidence of feature dominance between location and identity. What is the cause of different results in hearing, vision and haptics? Previous works interpreted non-mutual influences of identity and location as evidence of the primacy of certain attributes within specific sensory modalities [11], [16]. However, since the difficulty of the identity and the location tasks in some of the previous studies was not always equivalent (see [11], Experiments 3 and 4), we believe that the presence and direction of asymmetric cross-feature influences could be due to the characteristics of the specific tasks used to operationalize the features in analysis, rather than to modality-related feature dominances. Since we have not conducted cross-modal comparisons in our study, we can neither confirm nor rule out the presence of different binding mechanisms in haptics compared to hearing or vision.

Concerning the automaticity of binding, a cross-experiment comparison indicated that the sensitivity to variations in both location and texture is analogous to sensitivity to variation in either location or texture. Since binding does not have any negative effect on sensitivity, it appears that the integration of features is effortless and does not imply an increased cognitive load.

Which are the neural mechanisms subserving feature integration in working memory in haptics? As far as we know, there are no studies focusing on the neural mechanisms of “what” and “where” integration in haptics. However, previous research in the visual domain suggested a crucial role for the prefrontal cortex for the integration of spatial and non-spatial information [17], [8]. More specifically, a study by Munk and collaborators [18] compared the cortical activations associated with spatial, non-spatial and conjunction-working memory. They showed a distinction in location of cortical activation associated with spatial (more dorsal areas) and non spatial tasks (more ventral areas). Importantly, a what-and-where conjunction task resulted in activation of parts of both regions. It is important to point out that the what-and-where separation was not rigid. In fact, activation in the “where” dorsal prefrontal regions during the retention of “what” features and activations of “what” ventral prefrontal regions for “where” features were significantly higher than the baseline. This data suggests that a wide range of prefrontal areas are recruited during working memory regardless of the to-be-remembered characteristics and that the specific target feature modulates the activity of a distributed network [18]. Can these findings in the visual domain be extended to the haptic domain? Concerning haptic spatial processing, Kaas, van Mier, and Goebel [19] described a network of cortical areas which are active during the maintenance of information about the orientation of a haptic stimulus. They propose that haptic spatial information could be represented as abstract hapticospatial representations in a network involving principally the prefrontal and the parieto-occipital cortices. Such dorsal network is likely to be (at least partially) independent from the modality of the input. In fact, there is evidence that both the visual and tactile version of the same spatial task elicited neural responses in the dorsal “where” cortical pathway [20]. This evidence, among many others, supports a metamodal theory of neural computation according to which the cortex is specialized in types of computation, and that the sensory origin of the input does not matter [21]. Assuming that analogous supramodal mechanisms are also involved in the integration of “what” and “where” information in haptics, we could speculate that a wide network of prefrontal areas is active for tasks requiring the integration of “what” and “where” haptic information, similar to what was shown by Munk and colleagues in the visual domain [18]. Further research in this direction should be conducted to verify this assumption.

Finally, no effects of the contingent aspects of exploration, such as the direction of the exploration and the position of the items in space, suggest the intervention of recoding mechanisms, from an egocentric representation during exploration to an allocentric representation in maintenance and recall.

### Conclusion

The present study is the first study to provide evidence of binding between “what” and “where” information in WM for haptic stimuli. We investigated whether the representation of location and texture of tactile items is integrated in a conjunct representation both in a condition in which each is relevant for the task and in a condition where only the location or only the texture must be maintained in memory for future recall. When both features were task-relevant, results indicated that, even if the association of location and texture is neither necessary nor required to perform the task, participants integrate the two features in a single memory representation. By contrast, when only one feature is relevant for memory recall, results indicate that the task-irrelevant feature is not able to influence the recall of the target feature. Considering the combined result of the two experiments, we conclude that attention to the association between features is not necessary for the emergence of feature-to-location binding in haptic WM. For binding to take place, however, it is necessary to encode and maintain in memory both the identity and the location of items.
